# Origin of the Induced Pluripotent Stem Cells Affects Their Differentiation into Dopaminergic Neurons

**DOI:** 10.3390/ijms21165705

**Published:** 2020-08-09

**Authors:** Paula Chlebanowska, Maciej Sułkowski, Klaudia Skrzypek, Anna Tejchman, Agata Muszyńska, Rezvan Noroozi, Marcin Majka

**Affiliations:** 1 Jagiellonian University Medical College, Sw. Anny 12, 31-008 Kraków, Poland; paulalota0@gmail.com (P.C.); maciej.sulkowski@gmail.com (M.S.); klaudia.skrzypek@uj.edu.pl (K.S.); tejchman.anna@gmail.com (A.T.); 2 Department of Transplantation, Institute of Pediatrics, Jagiellonian University Medical College, Wielicka 265, 30-663 Krakow, Poland; 3 Bioinformatics Research Group, Malopolska Centre of Biotechnology, Jagiellonian University, Gronostajowa 7A, 30-387 Kraków, Poland; a.muszynska@uj.edu.pl; 4 Institute of Automatic Control, Electronics and Computer Science, Silesian University of Technology, Akademicka 16, 44-100 Gliwice, Poland; 5 Human Genome Variation Research Group, Malopolska Centre of Biotechnology, Jagiellonian University, Gronostajowa 7A, 30-387 Kraków, Poland; rezvan68noroozi@gmail.com

**Keywords:** origin of iPS cells, teratoma formation, differentiation, dopaminergic neurons, midbrain organoids

## Abstract

Neuronal differentiation of human induced pluripotent stem (iPS) cells, both in 2D models and 3D systems in vitro, allows for the study of disease pathomechanisms and the development of novel therapies. To verify if the origin of donor cells used for reprogramming to iPS cells can influence the differentiation abilities of iPS cells, peripheral blood mononuclear cells (PBMC) and keratinocytes were reprogrammed to iPS cells using the Sendai viral vector and were subsequently checked for pluripotency markers and the ability to form teratomas in vivo. Then, iPS cells were differentiated into dopaminergic neurons in 2D and 3D cultures. Both PBMC and keratinocyte-derived iPS cells were similarly reprogrammed to iPS cells, but they displayed differences in gene expression profiles and in teratoma compositions in vivo. During 3D organoid formation, the origin of iPS cells affected the levels of FOXA2 and LMX1A only in the first stages of neural differentiation, whereas in the 2D model, differences were detected at the levels of both early and late neural markers FOXA2, LMX1A, NURR1, TUBB and TH. To conclude, the origin of iPS cells may significantly affect iPS differentiation abilities in teratomas, as well as exerting effects on 2D differentiation into dopaminergic neurons and the early stages of 3D midbrain organoid formation.

## 1. Introduction

One of the major goals in neurobiology is to understand the development of the nervous system and to model cellular interactions within the human brain.

The introduction of specific genes encoding transcription factors, such as MYC, OCT3/4, SOX2 and KLF4, could reprogram human somatic cells into induced pluripotent stem (iPS) cells, and subsequently, iPS cells can be differentiated into any type of human cell [1]. Generated iPS cell lines can be characterized by the expression of endogenous pluripotency markers, activity of alkaline phosphatase and the capabilities of the cells to form embryoid bodies in vitro and teratomas in vivo [1,2,3]. The technology of generating iPS cells from somatic cells has opened new perspectives, such as cellular replacement, regenerative therapy and disease modeling [3].

Recently, the ability to generate neurons from human iPS cells has provided the opportunity to model the human brain. In the last decade, many protocols for generating nervous systems in 2D cultures in monolayer have been developed, which allow a high efficiency of differentiation [4,5]. Unfortunately, 2D models have limitations, as they do not show the organization of the nervous tissue and the interaction of the cells in the brain structure, which limits our understanding of complex processes such as embryonic development and tissue regeneration. In contrast to 2D models, organoids are three-dimension (3D) aggregates formed by different cell types that mimic the organization of brain structures. These 3D organoid models are self-organized structures that allow the observation of cellular interactions [6]. Both types of differentiation strategies are great tools, which can be used in various applications to study the pathomechanisms of different brain diseases, including Parkinson’s disease, Alzheimer’s disease, Huntington’s disease, autism spectrum disorder and schizophrenia [1,7,8,9,10,11].

It should be taken into consideration that the fate of iPS cells is guided by the expression of specific genes and epigenetic modulations, which may have an impact on further differentiation processes [12,13]. Generally, gene expression represents a current cell state, but the future differentiation potential can also be guided by epigenetics. It has been demonstrated previously that iPS cells retain their epigenetic memory of histone modifications, chromatin conformation and DNA methylation from the donor cell type. This epigenetic memory may exert an effect on the predisposition of iPS cells to differentiate [14,15,16,17,18]. Moreover, iPS cells may display differences in single clones, when pluripotency markers are evaluated [19,20].

The aim of this study was to examine whether the donor cell source can affect the reprograming and the further differentiation capabilities of iPS cells. Therefore, we generated several iPS clones from keratinocytes and peripheral blood mononuclear cells isolated from the same donor and evaluated the expression of endogenous pluripotency markers, the activity of alkaline phosphatase, as well as the gene expression and methylation profiles and capabilities of the cells to form embryoid bodies in vitro and teratomas in vivo. Subsequently, we characterized the capabilities of iPS cells to differentiate into 2D dopaminergic neurons and 3D midbrain like-organoids. Our studies suggest for the first time that the origin of iPS cells may be an important factor in the neural differentiation of iPS cells.

## 2. Results

### 2.1. Keratinocytes and Peripheral Blood Mononuclear Cells (PBMCs) Isolated from Healthy Donors Are Reprogrammed Similarly to Induced Pluripotent Stem (iPS) Cells with the Sendai Viral Vector, Whereas the Generated iPS Cells Display Different Gene Expression Profiles

Our main goal was to compare iPS cell lines generated from different cellular origins. Both keratinocytes and PBMCs seem to be very promising cell types for reprograming, as they can be easily isolated and efficiently reprogrammed [21]. The highest safety can be achieved using the Sendai viral vector, because it does not integrate into the genome [22,23].

**Figure 1:**
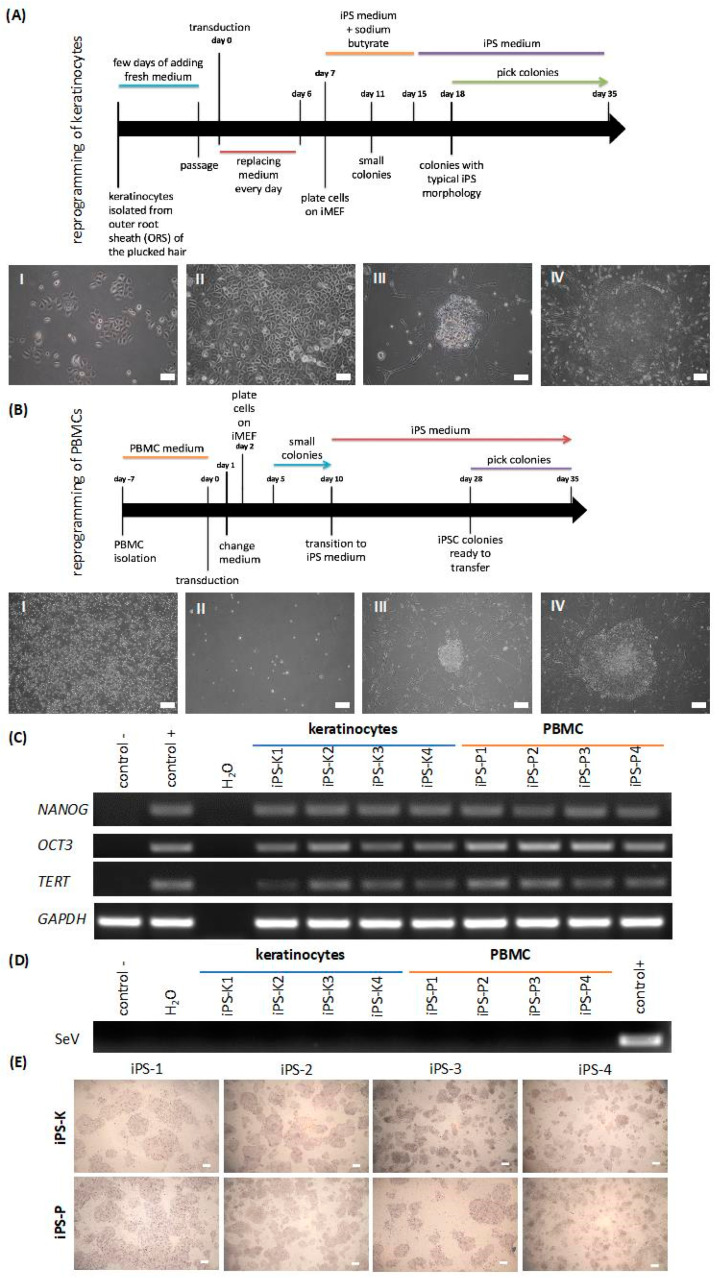
Reprogramming of keratinocytes and peripheral blood mononuclear cells (PBMCs) to induced pluripotent stem cells (iPSCs) with Sendai viral vector. Characterization involved 4 clones of each origin of iPS cells. (**A**) Scheme presenting generation of iPS cells from plucked hair keratinocytes. (**AI**) Representative image of the cells four days after transduction. (**AII**) Morphology of the tightly-packed cells, which is characteristic of the early stage of reprogramming. (**AIII**) Formation of the first colonies on day 14 after transduction. (**AIV**) A big and tightly packed colony, which is ready to be transferred, between 18 to 35 days. White scale bars represent 200 μm. (**B**) Scheme presenting generation of iPS cells from PBMCs. (**BI**) Representative image of PBMCs in the expansion medium five days before transduction. (**BII**) PBMCs one day after transduction with Sendai viral vector. (**BIII**) Formation of small colonies on day 16. (**BIV**) Big and tightly packed colony, ready to be transferred on day 28. White scale bars represent 200 μm. (**C**) Keratinocyte-derived-iPS (iPS-K) and PBMC-derived-iPS (iPS-P) cells express endogenous pluripotency genes, such as *NANOG*, *OCT3* and *TERT*. Positive control (control+) was a commercially available protein-induced iPS cell line (piPS), whereas negative control (control−) was a human colon cancer cell line, HTC116. (**D**) Viral transgene (SeV) was silenced in the generated cell lines after the 10th passage. Positive control for Sendai virus genome transgene was the generated iPS cell line at an early passage, whereas negative control was the piPS cell line. (**E**) Generated iPS clones displayed alkaline phosphatase activity. White scale bars represent 400 μm. Pictures show representative images. NANOG—homeobox protein NANOG; OCT3—octamer binding transcription factor 3; TERT—telomerase; GAPDH—housekeeping gene, glyceraldehyde 3-phosphate dehydrogenase; SeV—Sendai Virus genome.

To isolate keratinocytes, up to 20 hairs from a healthy volunteer were collected. After cell culturing of the hairs’ outer root sheath, a sufficient number of cells was acquired, within two to three weeks. Subsequently, the cells were transduced with the Sendai viral vector. Four days after transduction (Figure 1AI), the cells changed their morphology, a process which characterizes the early stage of reprogramming (Figure 1AII). The first colonies appeared on day 14, but numbers of colonies were not fully reprogrammed (pre-iPS) (Figure 1AIII). The fully reprogrammed keratinocyte-derived iPS (iPS-K) colonies, which were round and tightly packed, were detected around day 18. After 28 days, the colonies were ready to transfer (Figure 1AIV).

After isolation of PBMCs from whole blood, the cells were cultured in the expansion medium (Figure 1BI) and then, on day 0, they were transduced. On day 2, the morphology of the infected cells did not change (Figure 1BII). Between days 5 and 10 small colonies were noticeable (see Figure 1BIII). When iPS colonies were ready to transfer, their edges were regular and small cells were tightly packed (Figure 1BIV). Acquired PBMC-derived iPS (iPS-P) clones were expanded up to the 10th passage before further analysis.

Firstly, the presence of the Sendai virus genome was investigated. At the 10th passage, all the selected iPS lines were Sendai-virus-free, which suggested that viral transgenes (SeV) were silenced (Figure 1D). The iPS cell line, after reprogramming, at an early passage, was used as a positive control, whereas cancer cells (HCT116) were used as a negative control. The generated iPS cell lines expressed endogenous pluripotency markers at the mRNA level (Figure 1C), such as homeobox protein *NANOG*, octamer binding transcription factor 3 (*OCT3*) and telomerase (*TERT*). A commercially available protein-induced iPS (piPS) cell line was used a positive control, whereas cancer cells (HTC116) served as a negative control. Furthermore, all analyzed iPS clones displayed activity of alkaline phosphatase, as shown in Figure 1E.

**Figure 2:**
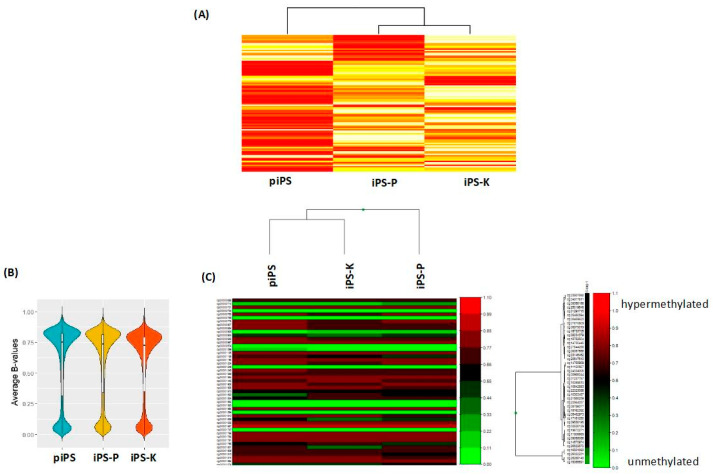
Analysis of transcription and methylation profile of iPS cells of different origin (**A**) Heatmap for mRNA expression microarray data after filtering and NEQC (normexp background correction and quantile normalization) normalization for 3 samples of iPS cells (piPS, iPS-K, iPS-P). Yellow color indicates genes displaying lower expression and red refers to genes with higher expression. (**B**) Violin plot showing the distribution of average methylation β-value for 3 samples (piPS, iPS-K, iPS-P). (**C**) Manhattan and correlation models were used to generate sample clustered heatmap of iPS cell lines (piPS, iPS-K, iPS-P) after methylation analysis. For cluster analysis, the most variable CpG probes were selected based on the s.d. of the β-values. The heatmap scale is from 0 to 1, where the green color indicates unmethylated sites and red refers to hypermethylated sites (β-value = 1).

After characterization of iPS cells, the transcription and methylation profiles were analyzed in the selected clones of iPS-K and iPS-P cells (Figure 2). The piPS cell line was our commercial control, generated from fibroblasts by protein introduction of transcription factors. The transcription profile, based on microarray analysis, showed differences for the 100 differentially expressed genes (Figure 2A). The violin plot (Figure 2B) showed that all three samples have similar methylation profiles. However, the methylation heatmap showed that iPS-P was clustered separately, compared to iPS-K and piPS samples (Figure 2C). All of the plots were created using average methylation beta values (0–1).

### 2.2. iPS Cells of Different Origin Form Embryoid Bodies and Three Germ Layers with Similar Effectiveness

**Figure 3:**
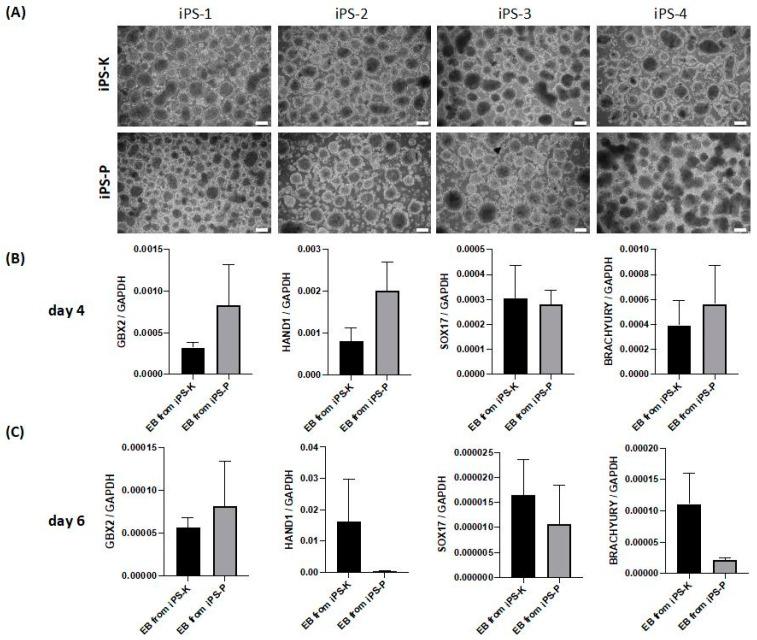
Formation of embryoid bodies from iPS cells. (**A**) Generated iPS cells formed embryoid bodies that displayed similar morphology (representative images). The photographs show representative images on day 4. White scale bars represent 200 μm. (**B**) Analysis of mRNA expression levels of markers of three germ layers in embryoid bodies on day 4: GBX2—ectodermal marker; HAND1—marker of early trophoblast differentiation; SOX17—endodermal marker; BRACHYURY—mesodermal marker. GAPDH was housekeeping gene control and the data were calculated using the ΔCt method. Significant differences between embryoid bodies (EBs) of different origin were not observed. The graph data show the results from 4 clones in duplicates, collected on day 4 (*n* = 8). The data represent the mean ± SEM. (**C**) Analysis of mRNA expression levels of markers of three germ layers in embryoid bodies on day 6. Significant differences between EBs of different origin were not observed on day 6. The graph data show the results from 3 clones, collected on day 6 (*n* = 3). The data represent the mean ± SEM.

To validate the effects of the different origins of cells on the capabilities of iPS cells to differentiate, embryoid bodies were formed from iPS cell lines. This is a common method for induction of differentiation towards various kinds of somatic cells and is a standard method used to validate iPS pluripotency [1,24,25]. All selected clones from both origins formed three-dimensional aggregates in suspension without any noticeable differences (Figure 3A).

Subsequently, markers of three germ layers and extraembryonic tissues (such as GBX2, HAND1, SOX17 and Brachyury) were investigated at the mRNA level (Figure 3B,C). Brachyury is a transcription factor in early mesodermal cells [26]. HAND1 is a transcription factor critical for specification of extraembryonic tissues (trophoblasts) [27,28]. SOX17 is a transcription factor that plays an important role in early endoderm development [29]. GBX2 is the early ectodermal lineages marker [30,31]. We observed large differences in the investigated genes between individual clones, which resulted in large variations within the groups. Nevertheless, no statistically significant differences between iPS-K and iPS-P were detected in the expression of selected markers on day 4 and 6 of differentiation (Figure 3B,C).

**Figure 4:**
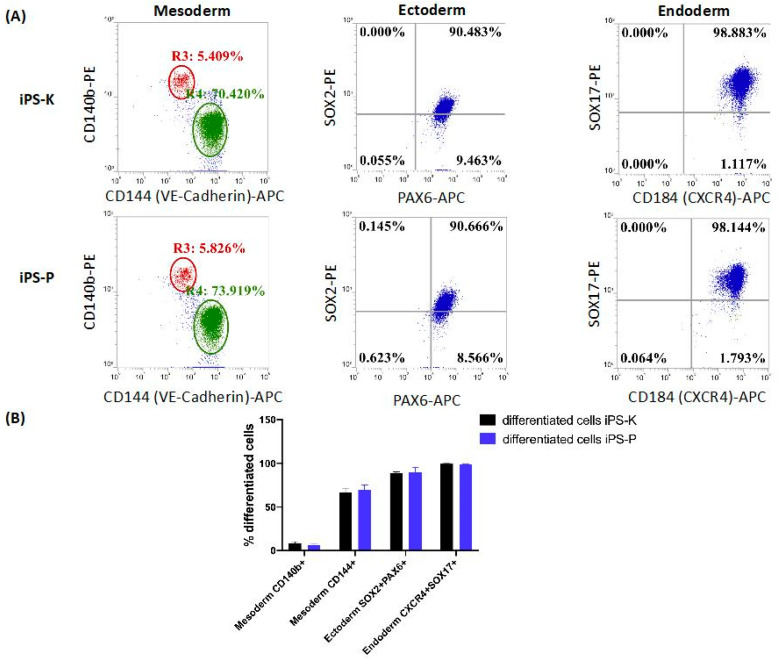
Differentiation iPS cells into three germ layers in vitro. (**A**) Representative plots of flow cytometry analysis of surface and intracellular marker expression of three differentiated iPS-K and iPS-P clones. The iPS cells were labelled with anti-CD144-PE, anti-140b-APC antibodies (mesodermal markers); anti-PAX6-APC, anti-SOX2-PE antibodies (ectodermal markers); anti-CD184-PE, anti-SOX17-APC antibodies (endodermal markers) and were analyzed by flow cytometry. (**B**) Graph presenting expression of various differentiation markers in three clones from iPS-K and three clones from iPS-P, *n* = 3. The results show mean +/− SEM.

Subsequently, markers of three germ layers (such as CD140b, CD144—mesoderm; SOX2, PAX6—ectoderm; SOX17, CD184—endoderm) were also investigated at the protein level after differentiation of iPS-K and iPS-P cells in vitro (Figure 4A). Flow cytometric analysis showed similar expression levels of the markers, characteristic of the first stage of differentiation into three germ layers for all three clones of iPS-K and three clones of iPS-P (Figure 4B). The analysis confirmed the RT-qPCR analysis performed on embryoid bodies. No significant differences were detected at the early stage of differentiation into three germ layers at the protein level.

### 2.3. Differentiation of iPS Cells in Teratomas Is Dependent on Origin of iPS Cells

**Figure 5:**
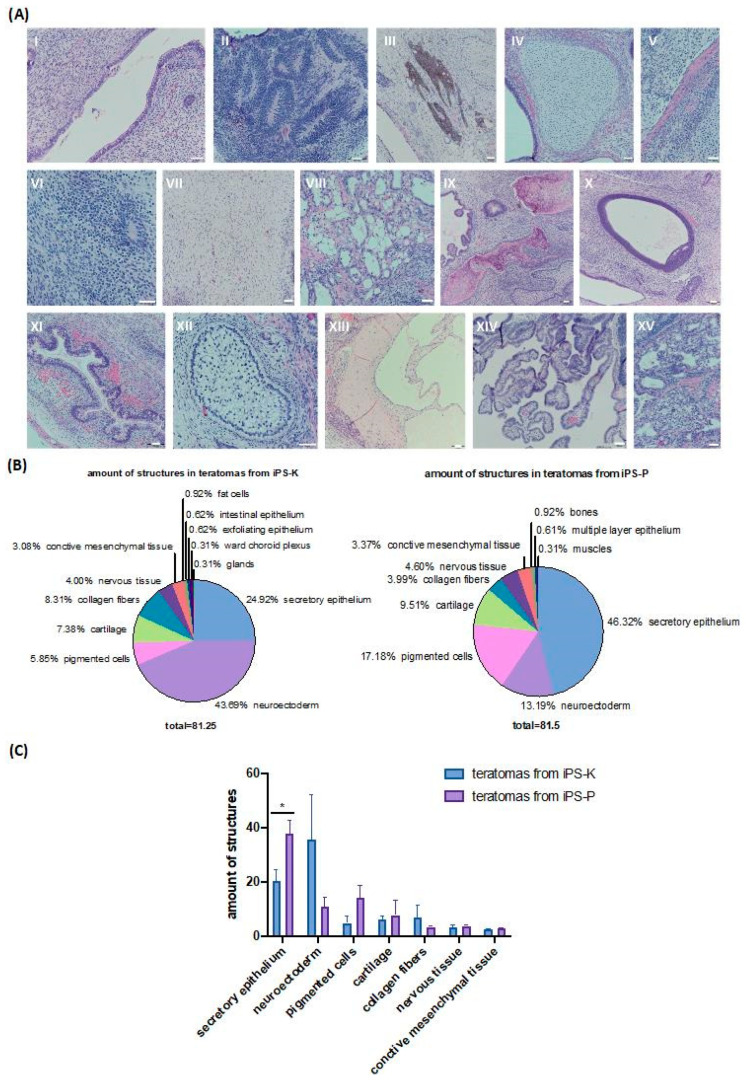
Formation of teratomas from iPS cells in vivo. (**A**) Representative image of structures which were found in the generated teratomas after subcutaneous implantation of iPS cells to immunodeficient NOD-SCID mice: (**AI**) secretory epithelium, (**AII**) neuroectoderm, (**AIII**) pigmented cells, (**AIV**) cartilage, (**AV**) collagen fibers, (**AVI**) nervous tissue, (**AVII**) connective mesenchymal tissue, (**AVIII**) fat cells, (**AIX**) bones, (**AX**) multiple layer epithelium, (**AXI**) intestinal epithelium, (**AXII**) exfoliating epithelium, (**AXIII**) muscles, (**AXIV**) ward choroid plexus, (**AXV**) glands. White scale bars represent 50 μm. (**B**) Pie charts showing average amounts of structures found in teratomas from iPS-K and iPS-P *n* = 4. (**C**) Graph presenting average amounts of structures in teratomas generated from iPS-K and iPS-P. Statistically significant higher amount of secretory epithelium was observed in teratomas from iPS-P (* *p* < 0.05), whereas a higher amount of neuroectoderm was noticed in teratomas generated from iPS-K. The photographs show representative images. The graph data represent the mean ± SEM.

The iPS-K and iPS-P cell lines were subjected to teratoma formation assays in immunodeficient NOD-SCID mice. Histopathological analysis of tumor slices enabled us to observe structures characteristic of all three germ layers within the tumors (Figure 5A). Subsequently, we analyzed the amount of tissue-specific structures in the generated teratomas (Figure 5B). We observed that in teratomas from iPS-K the most numerous structure was neuroectoderm, whereas in teratomas from iPS-P the most numerous structure was the secretory epithelium. The average amounts of the indicated structures in teratomas from four different clones between iPS-K and iPS-P are compared in Figure 5C. We also noticed that iPS-P-derived teratomas tend to form more structures of pigmented cells and cartilage. In iPS-K-derived teratomas, we observed a higher number of neuroectoderm-like structures and collagen fibers. Interestingly, structures characteristic of the mesoderm, such as bones and muscles, were detected only in teratomas generated from iPS-P.

These results confirm the efficient reprogramming of both keratinocytes and PBMC, and indicate the differences in the amount of three-germ-layer structures that could depend on the origin of iPS cells.

### 2.4. Origin of iPS Cells Affects Early Stages of Differentiation into 3D Midbrain Organoids

**Figure 6:**
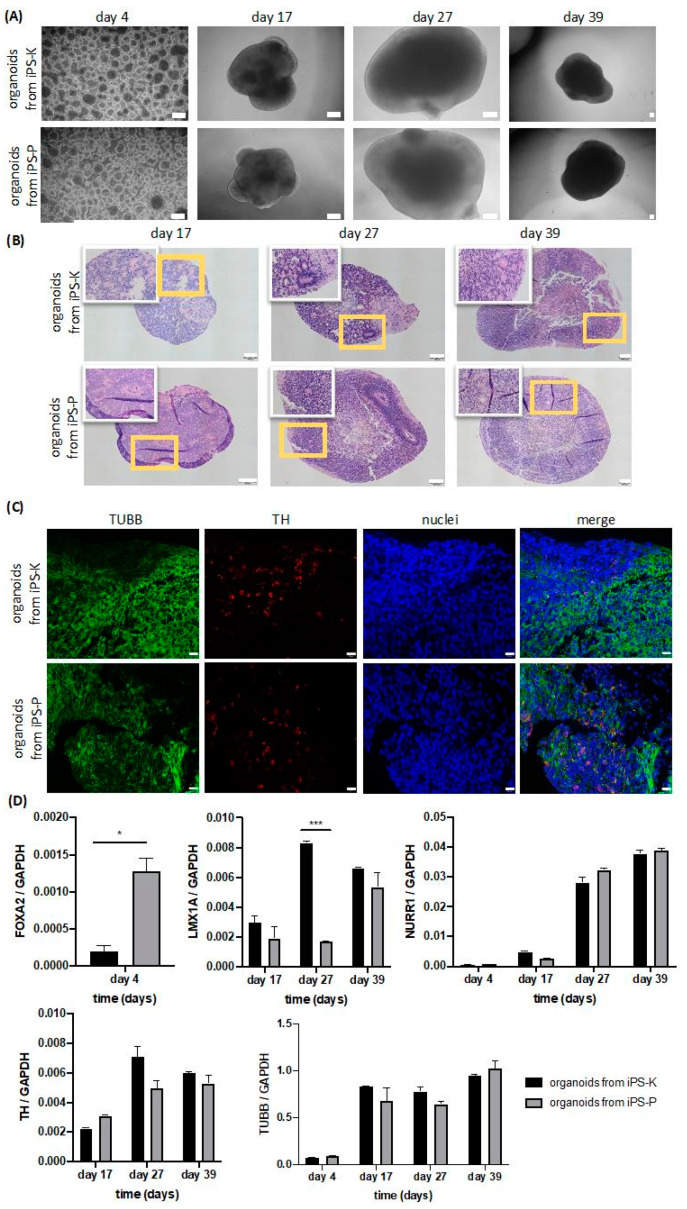
Differences in neuronal cell marker expression levels in organoids generated from iPS cells of differing origin. (**A**) Representative images presenting similar morphology of organoids during culture on days 4, 17, 27 and 39 (*n* = 4). White scale bars represent 200 μm. (**B**) Hematoxylin and eosin staining of organoids on day 17, 27 and 39 showed that organoids from iPS-K generated more structures resembling neuroectodermal rosettes. Yellow frame shows the selected structures in large magnification. Representative images are shown (*n* = 4). Scale bars represent 100 μm. (**C**) Representative images of immunofluorescent staining of TUBB and TH in organoids. On day 27 organoids from iPS cells generated from keratinocytes and PBMC contain TUBB+ and TH+ cells. White scale bars represent 20 μm. (**D**) mRNA expression levels of markers of neuronal progenitors (*FOXA2, LMX1A*) and markers of neurons (*NURR1, TH, TUBB*) in organoids from iPS-K and iPS-P. Representative data show results from 3 organoids (*n* = 3) in each timepoint. Statistically significant differences were observed in expression levels of *FOXA2* on day 4 and *LMX1A* on day 27. On day 4, higher expression of *FOXA2* was noted in organoids from iPS-P, * *p* < 0.05. On day 27, higher expression of *LMX1A* was observed in organoids from iPS-K, *** *p* < 0.001. Relative expression of genes was calculated by RT-qPCR with the ΔCt method, using GAPDH as a constitutive control. Statistically significant differences were analyzed using Student t-test. The experiments were performed using two clones of each type of iPS cells. Replicates for each clone were performed twice. In each experiment three organoids were collected for analysis. The data represent the mean ± SEM. TUBB—beta-III-tubulin, TH—tyrosine hydroxylase, NURR1—nuclear receptor related 1 protein, LMX1A—LIM homeobox transcription factor 1 alpha, FOXA2—forkhead box A2.

Midbrain organoids were generated according to the protocols of Jo. et al., 2016, with our previous modifications [1,6]. The morphology of both kinds of organoids was similar (Figure 6A), but the hematoxylin/eosin staining showed differences in their structures (Figure 6B). More neuroectodermal rosettes were observed in organoids generated from iPS-K in each timepoint (Figure 6D).

To further characterize the organoids, on day 27 we performed immunofluorescent staining for beta-III-tubulin (TUBB), which is a neuronal marker [32,33], and tyrosine hydroxylase (TH), which is a marker of dopaminergic neurons [34,35] (Figure 6C). We detected both TUBB and TH in both kinds of organoids. Furthermore, some of the TUBB-positive neurons were also positive for TH, which suggests that we were able to generate human midbrain organoids from both kinds of cells. Nevertheless, TH protein levels were similar in organoids generated from iPS-P and iPS-K cells (Figure 6C).

On days 4, 17, 27 and 39 we collected three organoids for evaluation of five markers at mRNA expression levels, such as LIM homeobox transcription factor 1 alpha (*LMX1A*), forkhead family of winged-helix transcription factor 2 (*FOXA2*), orphan nuclear receptor (*NURR1*), tyrosine hydroxylase (*TH*) and beta-III-tubulin (*TUBB*) (Figure 6D). Expression of the *FOXA2* gene was detected only on day 4 and was significantly higher in organoids from iPS-P cells (6.4-fold). On day 17, 27 and 39 expressions of *LMX1A* tended to be higher in organoids from iPS-K cells, but statistically a significant difference was observed only on day 27 (4.96-fold). From day 4 to day 39, the level of *NURR1* expression in both kinds of organoids was rising, and differences between origin of iPS were not detected. On day 17, 27 and 39 we detected expression of *TH* gene. On day 17 the level of *TH* expression was lower than on day 27 and 39. On day 27 and 39 *TH* gene was expressed at the same level in both groups of organoids. On day 4, level of *TUBB* was at the low level. Then on day 17, 27 and 39 levels of *TUBB* were similarly high and stayed approximately at the same level for both groups in all timepoints.

These results suggest that the origin of iPS cells may have an impact only on early stages of differentiation in 3D organoids, but not the whole differentiation process.

### 2.5. Origin of iPS Cells Affects Their Capability to Differentiate into 2D Dopaminergic Neurons

**Figure 7:**
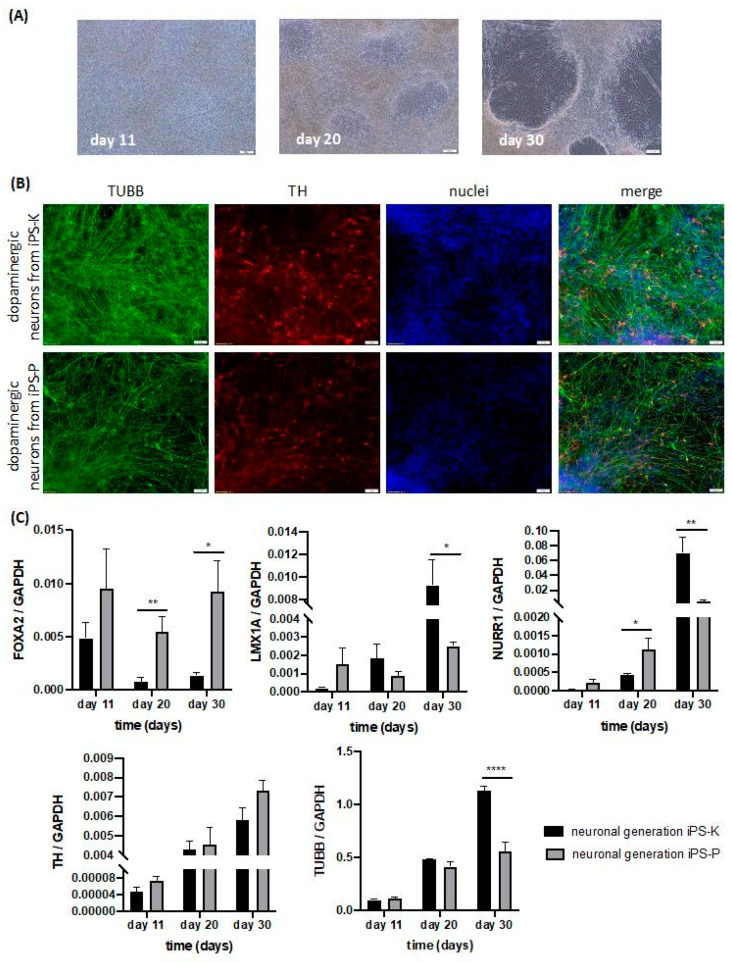
Differences in neuronal cell marker expression levels in dopaminergic neurons generated from iPS cells of different origin. (**A**) Representative images of morphology of 2D neural differentiation. White scale bars represent 100 μm. (**B**) Immunofluorescent staining of TUBB and TH in the generated dopaminergic neurons. On day 30, the differentiated cells from iPS-K and iPS-P cells contained TUBB-positive and TH-positive cells. Slightly higher expression of TH was observed in neurons generated from iPS-K cells. Representative images are shown. White scale bars represent 50 μm. (**C**) Gene expression of neuronal progenitors (*FOXA2, LMX1A*) and neurons markers (*NURR1, TH, TUBB*) in dopaminergic neurons generated from iPS-K and iPS-P in each timepoint (day 11, 20 and 30 of differentiation). Statistically significant higher expression of *FOXA2* gene was detected on day 20 and 30 in cells generated from iPS-P cells compared to iPS-K cells, ** *p* < 0.01, * *p* < 0.05. Higher expression of *LMX1A* was observed on day 30 in neurons generated from iPS-K, * *p* < 0.05. On day 20, statistically significant higher expression of the *NURR1* gene was observed in cells from iPS-P cells, but on day 30 higher expression of that gene was observed in neurons from iPS-K cells, * *p* < 0.05, ** *p* < 0.01. The expression of *TUBB* was definitely higher in neurons from iPS-K on the last day of differentiation and the differences were statistically significant, **** *p* < 0.0001. Relative expression of genes was calculated by RT-qPCR using the ΔCt method with *GAPDH* as constitutive control. Statistically significant differences were analyzed using Student’s t-test. The experiments were performed for two clones from every source of iPS cells. Replicates for each clone were performed twice (*n* = 4). The data represent the mean ± SEM. TUBB—beta-III-tubulin, TH—tyrosine hydroxylase, NURR1—nuclear receptor related 1 protein, LMX1A—LIM homeobox transcription factor 1 alpha, FOXA2—forkhead box A2.

Subsequently, we differentiated two selected iPS clones of each origin into dopaminergic neurons according to the 2D protocol described by Kriks et al., 2011 [5]. On day 11 neural progenitors were observed. Then, on day 20, cells with characteristic neuronal morphology started to appear. On day 30, mature dopaminergic neurons were detected (Figure 7A).

The presence of dopaminergic neurons was confirmed by TH expression at the protein level (Figure 7B). Therefore, staining for TH and TUBB was performed. Interestingly, the level of TH tended to be slightly increased in neurons generated from iPS-K cells (Figure 7B).

Furthermore, on days 11, 20 and 30, mRNA expression levels of *FOXA2*, *LMX1A*, *NURR1*, *TH* and *TUBB* were analyzed (Figure 7C). During midbrain differentiation, co-expression of the roof plate marker *LMX1A* and the floor plate marker *FOXA2* should be visible [5]. Cells from both iPS-K and iPS-P showed the expression of *FOXA2* on day 11, and then the expression decreased. On day 30, the increased levels of *FOXA2* in neurons generated from iPS-P were observed in contrast to neurons generated from iPS-K, as their level of *FOXA2* was similar to the level from day 20. Furthermore, a significantly higher level of *FOXA2* on day 20 (6.83-fold) and 30 (6.71-fold) was observed in cells from iPS-P cells. We found that, from day 11 to 30, the level of *LMX1A* in neural generation from iPS-K cells was rising. On day 30, we detected a significantly higher level of *LMX1A* (3.70-fold) in neurons from iPS-K, compared to neurons from iPS-P, whereas in cells generated from iPS-P, *LMX1A* expression on day 11 was higher than in cells generated from iPS-K, and then on day 20 it decreased, and on day 30 it increased again to the level from day 11. Cells from both groups showed increasing *NURR1* levels over time. On day 20 expression of *NURR1* was higher (2.63-fold) in cells generated from iPS-P, in contrast to day 30, when level of *NURR1* was significantly higher in neurons generated from iPS-K (13.14 fold). The mRNA level of *TH* increased over time and was similar in neurons generated from iPS cells of both origins. Level of *TUBB* also rose over time for both groups. On day 11 and 20 the same level of *TUBB* for neurons generated from iPS-K and iPS-P was observed, whereas on day 30 expression of *TUBB* gene was significantly higher (2.04-fold) in neurons generated from iPS-K.

These results demonstrate that during neural generation in a 2D model, differences at the mRNA and protein levels of neuronal markers may appear, depending on origin of iPS cells.

## 3. Discussion

Generating neurons in vitro provides a great opportunity to study neurodevelopment and pathogenesis of neurological diseases, and can be used in therapeutic approaches. Nowadays, many protocols for generating dopaminergic neurons in 2D models have been described. Efforts are being made to achieve the highest efficiency, standardization and reproducibility [4,5]. A new and also promising technique is generating 3D organoids, which consist of different kinds of cells apart from neurons, creating a more physiological environment for developing neurons. Three-dimensional neural tissue can mimic neurogenesis and the formation of particular neural structures [5,6]. Considering all applications of both models, their reproducibility is a very important feature. Our study examined whether the origin of iPS cells has an influence on their differentiation into neurons in 2D and 3D models, and, thus, on the reproducibility of the protocols.

The differences in transcriptional and epigenetic patterns in iPS cells of different origins, using a murine model, were firstly proposed in 2010 by Polo et al. [18]. Since that time, many publications have shown that iPS cells have epigenetic memory of their tissue of origin, which may affect their capability to differentiate [14,36,37]. On the other hand, there are also manuscripts suggesting that epigenetic memory has no impact on the generation of iPS cell lines from different cell origins [38], or indicating no impact of iPS origin on 2D differentiation to neuronal progenitor cells [39], so further research on that subject is still required. Our transcription and methylation analysis of iPS cells indeed showed differences between iPS cell lines of different origins. However, our other results demonstrated that at the level of basic characteristic of pluripotency features, there were no differences between iPS cells of different sources. Alkaline phosphatase staining, expression of endogenous pluripotency markers, embryoid body formation and differentiation into three-germ layers gave similar results for all investigated clones of iPS cell lines. Similarly in the literature, no significant changes have been observed in the basic characteristics of the iPS lines, both between clones of cells from one person and clones of the same source from different people [40,41]. Nevertheless, we noticed differences in the amount of structures characteristic for particular germ layers in the formed teratomas. More ectodermal structures were observed in teratomas formed from the keratinocyte-derived-iPS cell line, whereas some mesodermal structures, such as bones and muscles, were only detected in the PBMC-derived-iPS cell lines. These results suggest that the ectodermal origin of iPS cells may enhance their differentiation capabilities towards ectodermal structures. Importantly, the keratinocytes’ origin promoted the formation of higher numbers of neuroectodermal structures. Furthermore, in comparison to the literature, our method of analyzing teratoma structures is a novel and unique approach [42,43].

After the first signal that differences can appear in teratomas, further analysis included evaluation of iPS cells’ capabilities to differentiate into neurons. We selected two clones from every source of iPS cells, which displayed the highest variation between the selected structures in teratomas. Then, we started neural differentiation. Earlier research papers have also demonstrated that tissue of origin of iPS cells and their epigenetic signatures have an impact on differentiation preferences [13,14]. However, one paper suggested that it is not the cell type of origin, but rather, clones of iPS cells which may affect differentiation processes [44]. In our studies, we did not observe clonal differences in neuronal differentiation (data not shown), but we detected differences dependent on the origin of iPS cells. Both in 2D and 3D differentiation, we observed differences in gene expression levels during the process of neuron generation. These results are additionally supported by the higher levels of neuroectodermal structures formed in teratomas from keratinocytes-derived iPS cells.

An important aspect in the process of differentiation to dopaminergic neurons is the expression of neuronal progenitors’ markers. LMX1A and FOXA2 are early markers of midbrain floor plate progenitors [5]. FOXA2 is required for in vivo development of dopaminergic neurons, whereas LMX1A is crucial factor in the development of dopaminergic neurons, which also regulates the survival of adult dopaminergic neurons [45,46,47]. NURR1 is responsible for differentiation of early progenitors to mature dopaminergic neurons [48]. Moreover, the interaction of FOXA2 and NURR1 protects neurons against toxins [49].

In both neuronal differentiation types, we observed statistically significant differences in FOXA2 and LMX1A levels, which are markers of midbrain floor plate progenitors [5]. Therefore, we can conclude that the source of iPS cells may have a significant impact on the initial phase of neural differentiation in both 2D and 3D models.

In later stages of midbrain organoid formation, we did not observe any differences in gene expression. The 3D midbrain organoids displayed similar levels of TUBB- and TH-positive neurons. In contrast, on the last day of 2D differentiation to dopaminergic neurons, the expression of NURR1 and TUBB was definitely higher in neurons generated from keratinocyte-derived iPS cells. An important conclusion to draw from our data is that in 2D differentiation to dopaminergic neurons, the origin of iPS cells may also have an impact on the final results. The significant effects of the origin of iPS cells on their neuronal differentiation capabilities in the 2D model are in agreement with previous research showing distinct origin-dependent neural cell identities after 2D differentiation of iPS cells, despite the lack of differences in TUBB and TH protein levels [12].

In contrast to the 2D protocol, in 3D midbrain organoids, we did not observe any differences between organoids at the last stage of differentiation. The differences were visible only in the first steps of differentiation, which suggests that the cells of differing origins may have a distinct timeframe of differentiation, although the final result is the same.

In previous research, both 2D and 3D differentiation protocols for different structures were compared [50,51]. Nevertheless, in our work, we compared these two types of differentiation models in the context of the origin of iPS cells for the first time. In the 3D model we observed statistically significant differences only at the early stages of differentiation. It is possible that differentiation in structures resembling the natural environment in developing tissue may slightly attenuate the effect of epigenetic differences dependent on the origin of iPS cells, which was also suggested by other groups [12,52].

## 4. Materials and Methods

### 4.1. Cell Culture

Induced pluripotent stem (iPS) cells were generated from keratinocytes and PBMCs of the same donor, or were bought (piPS, generated from fibroblasts, SBI System Biosciences, Palo Alto, CA, USA). iPS cells were cultured in serum-free iPS medium containing DMEM/F12, 20% KSR, 2 mM Glutamax, 100 U/mL Penicillin/Streptomycin, 100 µM non-essential amino acids, 10 ng/mL bFGF (all from Thermo Fisher Scientific, Waltham, MA, USA) and 100 µM β-mercaptoethanol (Sigma-Aldrich, Saint Louis, MO, USA). iPS cells were cultured feeder free on Matrigel (Corning, New York, NY, USA) or on feeder layers of inactivated mouse embryonic fibroblasts (iMEFs) on dishes coated with gelatin (Sigma-Aldrich). Medium was changed every day. Routine passages were done using Accutase (Lonza, Basel, Switzerland). After the passage, iPS cells were seeded at a density of 1:4–1:10 in medium for iPS cells supplemented with 10 µM ROCK inhibitor Y-27632 (Sigma-Aldrich). Before freezing, iPS cells were incubated with 10 µM ROCK inhibitor for one hour on a culture dish. Then, iPS cells were suspended in freezing medium containing 90% fetal bovine serum (FBS; Eurx, Gdansk, Poland), 10% DMSO (Sigma-Aldrich) and 10 µM ROCK inhibitor (Sigma-Aldrich), frozen and cryopreserved in liquid nitrogen.

Keratinocytes were cultured in serum free EpiLife medium (Thermo Fisher Scientific) with 100 U/mL Penicillin/Streptomycin.

PBMCs were cultured in the expansion medium: serum free medium QBSF-60 (VWR, Radnor, PA, USA) supplemented with 10 µl/mL ascorbic acid (Thermo Fisher Scientific), 1.5 µM dexamethasone and growth factors: 50 ng/mL SCF, 10 ng/mL IL-3, 2 U/mL EPO, 40 ng/mL IGF-1 (all from PeproTech, London, United Kingdom) and 100 U/mL Penicillin/Streptomycin (Thermo Fisher Scientific).

Mouse embryonic fibroblasts (MEFs) (AMSBIO, Abingdon, United Kingdom) were cultured in Dulbecco’s modified Eagle’s medium (DMEM) containing 4.5 g/L glucose and supplemented with 10% FBS, 2 mM L-glutamine and 100 U/mL Penicillin/Streptomycin (all from Thermo Fisher Scientific). To generate the feeder layer for iPS culture, MEF cells were inactivated with mitomycin C (Sigma-Aldrich). To generate MEF-conditioned iPS medium, MEF medium was incubated for 24h on MEF cells. Then, medium was centrifuged at 2000 rpm for 10 min and supplemented with 10 ng/mL bFGF (Thermo Fisher Scientific).

HTC116 is a human colon colorectal cancer that was used as a negative control for pluripotency features. This cell line was cultured in Dulbecco’s modified Eagle’s medium (DMEM) containing 4.5 g/L glucose and supplemented with 10% FBS, 2 mM L-glutamine and 100 U/mL Penicillin/Streptomycin (all from Thermo Fisher Scientific). All cells were cultured at 37 °C in a humidified atmosphere and 5% CO₂ level.

### 4.2. Generation of iPS Cells from Keratinocytes and PBMC

The Jagiellonian University Bioethical Committee in Kraków approved the study using human samples-decision number KBET/173/B/2012 on 31 May 2012, with two prolongations from 17 December 2015 and 23 June 2016. Blood and hair were isolated from a healthy volunteer who gave informed consent to participate in the study.

PBMCs were isolated by centrifugation of blood on gradient of Pancoll (PAN Biotech, Aidenbach, Germany), as described previously [1]. The acquired layer of mononuclear cells isolated from blood was washed three times with Phosphate Buffered Saline (PBS, Eurx). PBMCs were cultured in the expansion medium, described previously [1]. After 7 days, PBMCs were transduced with the Sendai viral vector from CytoTune^®^ 2.0 Sendai Reprogramming Kit (Thermo Fisher Scientific). Forty-eight hours after transduction, the cells were plated on inactivated mouse embryonal fibroblasts (iMEF). The first colonies (pre-iPS) appeared between 5 and 10 days. Mature colonies, ready to transfer, appeared around day 28. After 28 days, the colonies were picked and transferred to separate wells. After the 10th passage, iPS lines were cryopreserved and tested.

Keratinocytes were isolated from the outer root sheath (ORS) of the plucked hair, according the the protocols of Aasen et al., 2010, with modifications described previously by Sulkowski et al., 2018 [2,40]. The isolated hairs were rinsed with antimycotic-antibiotic solution (Thermo Fisher Scientific). Then, the hairs were cut and placed in a dish with coated with Matrigel (Corning) in MEF-conditioned iPS medium. The medium was changed until keratinocytes were surrounding the hair. Then, medium was changed to EpiLife (Thermo Fisher Scientific) and cells were cultured on dishes coated with Matrigel. For the dissociation of the cells, TrypLE solution (Thermo Fisher Scientific) was used. Keratinocytes displaying 40–60% confluence were reprogrammed using the Sendai viral vector-CytoTune^®^ 2.0 Sendai Reprogramming Kit (Thermo Fisher Scientific), as described previously [2]. Twenty-four hours after transduction, the medium was changed. Seven days after transduction, cells were plated on iMEF in iPS medium with 1 mM sodium butyrate (Sigma-Aldrich). Sodium butyrate was added to the medium until day 8. On day 11, the first iPS (pre-iPS) cell colonies were detected. Between days 18 and 35, iPS colonies were picked and transferred to separate wells in iPS medium with 10 µM ROCK inhibitor Y-27632 (Sigma-Aldrich). After the 10th passage, iPS cells were ready for further experiments.

### 4.3. RNA Isolation and Reverse Transcription

RNA was isolated using the Universal RNA/miRNA Purification Kit (Eurx), according to vendor’s protocol. RNA was reverse transcribed to cDNA using the Moloney Murine Leukemia Virus reverse transcription kit (M-MLV Reverse Transcriptase, Promega, Madison, WI, USA), according to vendor’s protocol.

### 4.4. Polymerase Chain Reaction (PCR) Analysis

PCR reaction was performed using primers with optimal annealing temperature of 55 °C and Taq PCR Master Mix (Eurx). The following primers (5′ to 3′) were used: NANOG forward TGAACCTCAGCTACAAACAG, NANOG reverse TGGTGGTAGGAAGAGTAAAG, OCT3 forward ATGGCGGGACACCTGGCTT, OCT3 reverse GGGAGAGCCCAGAGTGGTGACG, TERT forward TGTGCACCAACATCTACAAG, TERT reverse GCGTTCTTGGCTTTCAGGAT, GAPDH forward CAAAGTTGTCATGGATGACC, GAPDH reverse CCATGGAGAAGGCTGGGG. Primers for the Sendai virus genome (SeV) were provided in the Sendai Viral Vector Kit.

### 4.5. Quantitative Real-Time PCR

The reaction was performed using: Blank qPCR Master Mix 2× (Eurx) and the TaqMan Expression Assays (Thermo Fisher Scientific). For embryoid bodies: *GBX2* Hs00230965_m1, *HAND1* Hs02330376_s1, *SOX17* Hs00751752_s1 and *Brachyury* Hs00610080_m1 (Thermo Fisher Scientific). For organoids and neurons: *LMX1A* Hs00898455_m1, *FOXA2* Hs00232764_m1, *NURR1* Hs01117527_g1, *TH* Hs00165941_m1 and *TUBB* Hs00801390_s1 (Thermo Fisher Scientific). The reaction was carried out using Quant Studio 7 System (Applied Biosystems, Foster City, CA, USA). The levels of mRNA expression of the analyzed genes were normalized to the housekeeping gene *GAPDH* (Hs02758991_g1), using the 2^−ΔCt^ method.

### 4.6. Alkaline Phosphatase (AP) Activity

iPS cells were fixed in 4% paraformaldehyde for 10 min. After fixation, the cells were washed three times with PBS and incubated in AP staining solution NBT-BCIP (Roche, Basel, Switzerland), 5 M NaCl, 1 M Tris-HCl (pH 9.5), 1 M MgCl_2_ for 10 min at room temperature in the dark. At the end, the cells were washed two times. The stained cells were analyzed using an IX70 microscope (Olympus Corporation, Tokyo, Japan) and images were collected using CellSensDimension software.

### 4.7. Gene Expression and Methylation Quantification

After isolation, RNA quality and concentration from all samples (3 samples: piPS, iPS-K and iPS-P) were checked on an Agilent TapeStation System (Agilent Technologies, Santa Clara, CA, USA) and a Promega QuantiFluor Dye System on a Quantus Fluorometer (Promega). To generate biotinylated cRNA from 300 ng of isolated RNA, the TargetAmp-Nano Labeling Kit for Illumina Expression BeadChip (Epicentre-an Illumina Company, Madison, WI, USA) was used. Prepared probes were fragmented and hybridized to an Illumina Whole Genome Expression Chip, HumanHT-12 v3.0. Then, BeadChips were washed and subsequently scanned. Gene expression profiles were made from 750 ng hybridized cRNA to Illumina HumanHT-12 v3.0 BeadChips. The hybridization assay was performed according to the Illumina whole-genome gene expression hybridization assay guide (Illumina, San Diego, CA, USA).

The heatmap was made after NEQC normalization and poor-quality samples were filtered out. Results were obtained for the 100 most variable genes.

The DNA methylation analysis was isolated by means of a QIAGEN QIAamp DNA Mini-Kit (QIAGEN, Hilden, Germany). The methylation assay was made according to the Infinium HD Methylation Assay Protocol Guide manual workflow for the Infinium Methylation BeadChips (Illumina)

The data for 3 samples (piPS, iPS-K and iPS-P) were analyzed and visualized using GenomeStudio software (Methylation Module v1.8). Detected CpGs (0.05) for all samples were >95%. The methylation status of each CpG site is reported as the average B-value (0–1) which is the proportion of the methylated signal to the total signal, with a small constant added to the denominator for stabilization. For methylated CpG, signal_B is high and signal_A is low, then the calculated β is near 1 (the ratio of methylated to total signal). Hypomethylated probes (β values < 0.2) and hypermethylated probes (β values > 0.7) can be interrogated and compared across samples for large-scale studies. Raw data for average B-values and intensities for each CpG per sample were calculated. The Illumina internal controls and background subtraction were applied to the samples. All three samples passed the quality control steps.

### 4.8. Formation of Embryoid Bodies

For dissociation, feeder-free iPS cells were incubated with 1 U/mL dispase (Thermo Fisher Scientific) for 5–10 min. The cells were then washed with PBS with Mg and Ca (Eurx). Cell clumps were gently collected to new tubes and centrifuged at 300 rpm for 3 min. The aggregates of cells were suspended in iPS medium without bFGF (Thermo Fisher Scientific). The medium was changed every day. After 4 days, embryoid bodies were ready for further experiments or analysis. The representative pictures on day 4 were shown. Analysis was also performed on day 6. The graph data show the results from 4 clones in duplicate, collected on day 4 (*n* = 8) and 3 clones (*n* = 3) on day 6.

### 4.9. Differentiation to Three Germ Layers

Functional characterization of iPS cell lines was performed using a StemMACS Trilineage Differentiation Kit (Miltenyi Biotec, Bergisch Gladbach, Germany). Each cell line was seeded on 3 wells of 24-well plate. The media were changed according to the Differentiation Kit Protocol. After 7 days, cells were passaged and counted for further analysis.

### 4.10. Staining and Flow Cytometry

Expression of specific markers of the mesoderm, ectoderm and endoderm were analyzed according to the StemMACS Trilineage Differentiation Kit (Miltenyi Biotec, Bergisch Gladbach, Germany). Five hundred thousand cells were stained with antibodies. For staining of surface markers, the cells were alive, whereas for intracellular staining, the cells were fixed by BD Cytoperm/Cytofix Plus (Becton Dickinson, Franklin Lakes, NJ, USA). The differentiated cells were incubated with fluorochrome-conjugated antibodies (fluorescein allophycocyanin-APC and phycoerythrin-PE): anti-CD144-PE, anti-CD140b-APC (mesodermal markers); anti-PAX6-APC, anti-SOX2-PE (ectodermal markers); and anti-CD184-PE and anti-SOX17-APC (endodermal markers). According to the protocol, the negative controls were the unstained cells. The staining was analyzed using Attune NxT Software v2.2 on the Attune Nxt Flow cytometer (Thermo Fisher Scientific).

### 4.11. Formation of Teratomas

All experimental protocols on NOD-SCID mice were approved by the 2nd Local Institutional Animal Care and Use Committee (IACUC) in Cracow, decision numbers: 162/2015 and 11/2018. iPS cells were passaged by Accutase (Lonza), centrifuged and suspended in Matrigel (Corning) and PBS (Eurx) in proportions of 1:1. Two hundred microliters (2 × 10^6^ cells) of the suspended cells were injected into the left dorsal flank of an adult female non-obese diabetic/severe combined immunodeficiency (NOD-SCID) mouse. Three mice were injected with each iPS clone; four iPS clones of each origin of iPS cells were tested. Tumor growth and mice health were controlled every day. Two months after the injection, teratomas were excised. Hematoxylin-eosin (H/E) staining was performed, as described previously [1]. After the staining, the amount of different structures in the tumor were analyzed. After characterization of particular structures, their amount was counted for all analyzed teratomas from clones from iPS-K and iPS-P. The structures were counted from 4 independent preparations for each clone. The counted structures were assessed by an experienced pathomorphologist. Graphs present the average amounts of structures in teratomas generated from iPS-K (generated from keratinocytes) and iPS-P (generated from PBMC); *n* = 4. Pie charts represent the average amount of structures in all analyzed teratomas from particular types of iPS origin. Bar graphs compare the average amount of particular structures between both types of teratomas from different iPS origins.

### 4.12. Formation of Midbrain Organoids

The protocol of generating midbrain organoids was previously described by Jo et al., 2016, and modified by Chlebanowska et al., 2020 [1,6]. Human iPS cells were dissociated into small cell aggregates, which were cultured for four days to form embryoid bodies (EBs). After this time, 48 EBs were individually transferred to a V-shaped 96-well low binding plate. Neuroectodermal differentiation was promoted by synchronous addition of dual-SMAD inhibition factors, like SB431542 or Noggin, and Wnt pathway activators, like CHIR99021, to the culture medium [6]. After transfer of EBs, they were cultured in DMEM F12/Neurobasal 1:1 (Thermo Fisher Scientific), which was supplemented with N2 1:100 (Thermo Fisher Scientific), 1:50 B27-vitamin A (Thermo Fisher Scientific), 100 µM non-essential amino acids (Thermo Fisher Scientific), 0.1% β-mercaptoethanol (Sigma-Aldrich), 1 µg/mL heparin (Sigma-Aldrich), 10 µM SB 431542 (Sigma-Aldrich), 200 ng/mL Noggin (PeproTech), 0.8 µM CHIR 99021 (Sigma-Aldrich), 100 U/mL Penicillin/Streptomycin (Thermo Fisher Scientific) for 4 days to start the formation of organoids. Mesencephalic fate was promoted by the addition of FGF8 and Sonic hedgehog, as suggested by Jo et al., 2016 [6]. Therefore, the organoids were cultured in previously described medium containing 100 ng/mL FGF8 (PeproTech) and 100 ng/mL SHH-C25II (PeproTech) for 3 days. Afterwards, each neuroectodermal structure was incubated in Matrigel (Corning) for 30 min to promote organization and growth in the 3D structure. The spheroids were cultured for 24 h in Neurobasal medium (Thermo Fisher Scientific) containing Matrigel (Corning) and supplemented with 1:100 N2 (Thermo Fisher Scientific), 1:50 B27-vitamin A (Thermo Fisher Scientific), 100 µM non-essential amino Acids (Thermo Fisher Scientific), 0.1% β-mercaptoethanol (Sigma-Aldrich), 1% glutamax (PAN Biotech), 2.5 µg/mL insulin (Sigma-Aldrich), 200 ng/mL laminin (Thermo Fisher Scientific), 100 U/mL Penicillin/Streptomycin (Thermo Fisher Scientific), 100 ng/mL SHH-C25II (PeproTech) and 100 ng/mL FGF-8 (PeproTech), and then they were transferred to a separate low binding plate with organoid growth medium, consisting of neurobasal medium (Thermo Fisher Scientific) supplemented with 10 ng/mL BDNF (PeproTech), 10 ng/mL GDNF (PeproTech), 100 µM ascorbic acid (Sigma-Aldrich) and 125 µM db-cAMP (Sigma-Aldrich). Starting from day 11, the spheroids were persistently cultured on orbital shaker until day 39 in organoid growth medium. The medium was changed every 3 days. To generate organoids, two clones from both kinds of iPS cell lines were selected. All experiments were repeated two times and in each analysis 3 organoids were used.

### 4.13. Differentiation of iPS Cells to Dopaminergic Neurons

The protocol of generating dopaminergic neurons was previously described by Kriks et al., 2011 [5,50]. iPS cells were plated at a density of 3.5–4 × 10^4^ cells/cm^2^ on Matrigel (Corning) in DMEM high glucose medium, supplemented with 15% knockout serum replacement (KSR), 2 mM L-glutamine, 10 μM β-mercaptoethanol and 100 U/mL Penicillin/Streptomycin (all from Thermo Fisher Scientific) with supplements LDN193189 (100nM, Stemgent, Cambridge, MA, USA), SB431542 (10 μM, Sigma-Aldrich). In the next two days, the following supplements were added to the medium from day 0: 100 ng/mL SHH C25II (PeproTech), 2 μM Purmorphamine (Stemgent) and 100 ng/mL FGF8 (PeproTech). On days 3 and 4, 3 μM CHIR99021 (CHIR; Stemgent) was added to the medium. From day 5, the KSR medium was gradually replaced with N2 medium, using the following proportions of KSR to N2: day 5 and 6—3:1; day 7 and 8—1:1; day 9 and 10—1:3. N2 medium consisted of DMEM/F12, N2 supplement and 100 U/mL Penicillin/Streptomycin (all from Thermo Fisher Scientific). From days 5 to 10, the medium was supplemented with 3 μM CHIR99021 (CHIR, Stemgent) and 100 nM LDN193189 (Stemgent). On days 5 and 6 the following supplements were added to the medium: 100 ng/mL SHH C25II (PeproTech), 2 μM Purmorphamine (Stemgent), 100 ng/mL FGF8 (PeproTech). From days 11 to 19, the medium consisted of Neurobasal Plus, B27 Plus supplement, 20 ng/mL BDNF (brain-derived neurotrophic factor, PeproTech), 0.2 mM ascorbic acid (AA, Sigma-Aldrich), 20 ng/mL GDNF (glial cell line-derived neurotrophic factor, PeproTech), 1 ng/mL TGFβ3 (transforming growth factor type β3, PeproTech), 0.5 mM dibutyryl cAMP (Sigma-Aldrich), 2 mM L-glutamine (Thermo Fisher Scientific) and 100 U/mL Penicillin/Streptomycin (Thermo Fisher Scientific). On days 11 and 12, 3 μM CHIR99021 (CHIR, Stemgent) was added to the medium. On day 20, the cells were dissociated using Accutase (Lonza) and seeded at high density (3–4 × 10^5^ cells/cm^2^) on an earlier plate coated with 15 μg/mL polyornithine (PO), 1 μg/mL laminin and 2 μg/mL fibronectin. From day 20, the medium consisted of Neurobasal Plus, B27 Plus supplement, 20 ng/mL BDNF (brain-derived neurotrophic factor, PeproTech), 0.2 mM ascorbic acid (AA, Sigma-Aldrich), 20 ng/mL GDNF (glial cell line-derived neurotrophic factor, PeproTech), 1 ng/mL TGFβ3 (transforming growth factor type β3, PeproTech), 0.5 mM dibutyryl cAMP (Sigma-Aldrich), 100 U/mL Penicillin/Streptomycin (Thermo Fisher Scientific) and 10 nM DAPT (Stemgent). The medium was changed every day, up to the desired point of neuron formation. The experiments were performed for two clones of every source of iPS cells. Replicates for each clone were performed twice (*n* = 4).

### 4.14. Immunofluorescent Staining

Organoids were fixed in 4% paraformaldehyde for 24 h at room temperature. After cell fixation, paraffin blocks were prepared in the Pathology Laboratory of the Children Hospital in Cracow. Three-micrometer slides were washed twice in Tris-buffered saline (TBS, 50 mM Tris-Cl, pH 7.6; 150 mM NaCl) containing 0.025% Triton X-100 (Sigma-Aldrich) for 5 min. Next, organoids slides were blocked for 1 h at room temperature in TBS containing 1% bovine serum albumin (BSA, Sigma-Aldrich). Then, slides were incubated with primary antibodies for 1 h: rabbit anti-tyrosine hydroxylase antibody (TH AB152, Merck-Millipore, CA, USA) and mouse anti-tubulin antibody, beta III isoform (TUBB MAB1637, Sigma-Aldrich). Subsequently, organoids slides were washed twice in TBS containing 0.025% Triton X-100 (Sigma-Aldrich) for 5 min. Then, the goat anti-rabbit and anti-mouse antibodies, conjugated with Alexa Fluor 555 (Thermo Fisher Scientific) and with addition of Hoechst (Sigma-Aldrich) in 1% BSA in TBS, were added to the slides and incubated at room temperature for 1 h in the dark. After the incubation, the slides were washed and covered in mounting medium (DAKO, Glostrup, Denmark) with a cover glass. The experiments were performed using two clones of each type of iPS cells. Replicates for each clone were performed twice. In each experiment three organoids were collected for analysis.

Differentiated neurons in the 2D model were washed in PBS (Eurx) and fixed in 4% paraformaldehyde at room temperature for 20 min. Then, the washed cells were incubated with 0.01% Triton X-100 at room temperature for 5 min. Subsequently, the cells were washed in PBS (Eurx) and blocked in 3% bovine serum albumin (BSA, Sigma-Aldrich) at room temperature for 30 min. Then, neurons were incubated with primary antibodies: rabbit anti-tyrosine hydroxylase antibody (TH AB152, Merck-Millipore) and mouse anti-tubulin antibody, beta III isoform (TUBB MAB1637, Sigma-Aldrich) in 3% BSA for 24 h at 4 °C, diluted. Subsequently, neurons were washed and secondary antibodies (goat anti-rabbit antibodies conjugated to Alexa Fluor 555 and anti-mouse antibodies conjugated to Alexa Fluor 488 (Thermo Fisher Scientific) and Hoechst (Sigma-Aldrich) in 3% BSA were added. After 1 h incubation in the dark, the cells were washed and covered in mounting medium (DAKO, Glostrup, Denmark) with cover glasses. The experiments were performed for two clones of every source of iPS cells. Replicates for each clone were performed twice (*n* = 4).

### 4.15. Statistical Analysis

GraphPad Prism version 8.4.2 was used to analyze the acquired data. To analyze statistically significant differences between two compared groups, Student’s t-test was performed. Data on graphs were presented as means ± SEM. Statistically significant differences (*p* < 0.05) are represented in graphs by the * symbol.

## 5. Conclusions

To conclude, our findings suggest that the origin of iPS cells may significantly affect their differentiation capabilities in different models, such as differentiation in teratomas in vivo, as well as exerting effects in models of both 2D and 3D differentiation into dopaminergic neurons in vitro. Keratinocyte-derived iPS cells may display a bias towards the formation of a higher number of neuroectodermal structures in teratomas in vivo and increased expression levels of different neural factors in 2D and 3D differentiation into dopaminergic neurons in vitro. These limitations should be taken into consideration in the design of good models of different neuronal diseases, such as Parkinson’s disease and Alzheimer’s disease [1,53].

## Abbreviations

iPSCs	Induced pluripotent stem cells
iPS-K	Induced pluripotent stem—keratinocytes
iPS-P	Induced pluripotent stem—PBMCs
PBMCs	Peripheral blood mononuclear cells
EB	Embryoid bodies
MEFs	Mouse embryonic fibroblasts
SeV	Sendai viral vector
